# Reinforcing or Disrupting Gender Affirmation: The Impact of Cancer on Transgender Embodiment and Identity

**DOI:** 10.1007/s10508-023-02530-9

**Published:** 2023-01-23

**Authors:** Jane M. Ussher, Rosalie Power, Kimberley Allison, Samantha Sperring, Chloe Parton, Janette Perz, Cristyn Davies, Teddy Cook, Alexandra J. Hawkey, Kerry H. Robinson, Martha Hickey, Antoinette Anazodo, Colin Ellis

**Affiliations:** 1 Translational Health Research Institute, School of Medicine, Western Sydney University, Locked Bag 1797, Penrith, Sydney, NSW 2752 Australia; 2 School of Health, Te Herenga Waka – Victoria University of Wellington, Wellington, New Zealand; 3 Specialty of Child and Adolescent Health, Faculty of Medicine and Health, University of Sydney, Camperdown, Sydney Australia; 4 School of Social Sciences, Western Sydney University, Penrith, Sydney Australia; 5 TransHub, ACON, Surry Hills, Sydney Australia; 6 School of Social Sciences and Translational Health Research Institute, Western Sydney University, Sydney, Australia; 7 Department of Obstetrics and Gynaecology, University of Melbourne and the Royal Women’s Hospital, Melbourne, Australia; 8 Kids Cancer Centre, Sydney Children’s Hospital and School of Women’s and Children’s, University of New South Wales, Sydney, Australia

**Keywords:** Cancer, Transgender, Embodiment and identity, Healthcare professionals

## Abstract

There is a pressing need for greater understanding and focus on cancer survivorship and informal cancer caring of trans people (binary and non-binary), across tumor types, to inform culturally safe trans inclusive cancer information and care. This qualitative study, part of the mixed methods Out with Cancer project, examined experiences of trans embodiment and identity after cancer diagnosis and treatment. We drew on open-ended survey responses from 63 trans cancer survivors and 23 trans cancer carers, as well as interviews and a photo-elicitation activity with a subset of 22 participants (15 cancer survivors, 7 cancer carers). Reflexive thematic analysis identified three themes: Cancer enhances trans embodiment, through experiences of gender euphoria following cancer treatment, and acceleration of decisions about gender affirmation; cancer erases or inhibits gender affirmation; trans embodiment is invisible or pathologized in cancer care. These findings demonstrate that trans embodiment and identity, as well as the process of gender affirmation, may be disrupted by cancer or informal cancer caring. Conversely, cancer and cancer treatment can positively impact the embodied identity and lives of trans people, despite the anxiety and strain of negotiating medical procedures. However, if healthcare professionals operate within a cis-heteronormative framework and do not understand the meaning of embodied change following cancer treatment for trans individuals, these positive benefits may not be realized.

## Introduction

There is a pressing need for attention to be paid to the cancer survivorship and cancer caring experiences of trans people (binary and non-binary) to inform culturally safe cancer information and care (Pratt-Chapman et al., 2021). Research in the emerging area of lesbian, gay, bisexual, trans, queer and intersex (LGBTQI) cancer survivorship primarily focuses on cisgender (cis) individuals (Taylor & Bryson, 2016), with few trans people included in study samples (Pratt-Chapman et al., 2021). Trans people may share many of the experiences of the broader lesbian, gay, bisexual, queer (LGBQ) community, including negative impacts of minority stress, and concerns about invisibility and discrimination in cancer care (Lisy et al., 2018; Quinn et al., 2015). However, trans people also have unique needs and experiences concerning cancer survivorship and care, which need to be acknowledged and addressed, in order to inform trans inclusive cancer care (Squires et al., 2022).

Until recently, investigation of cancer in trans communities has predominantly focused on epidemiology, etiology and biomedical aspects of cancer treatment (Watters et al., 2015), or involved medical case studies (Watters et al., 2015). There are reports of a higher prevalence of cancer amongst trans men, relative to cis men, attributed to higher rates of HPV and HIV infection, smoking and alcohol use, and medical comorbidities (Boehmer et al., 2020; Grant et al., 2011; Stutterheim et al., 2021). Exogenous hormone use as part of gender affirmation has been considered a cancer risk factor (Kerr et al., 2019). In the largest cohort study of trans people in Europe, trans women on gender affirmation hormone therapy (GAHT) were observed to have a higher risk of breast cancer than cis men but a lower risk compared with cis women (Jackson et al., 2022). However, longer-term studies suggest exogenous hormones may not be a cancer risk factor for trans people (Brown & Jones, 2015; Taylor & Bryson, 2016), and there is agreement on the pressing need for longitudinal research in this sphere (Jackson et al., 2022).

Trans populations may experience significant complications in the cancer diagnostic process due to socioeconomic issues, including higher rates of poverty and lower rates of health insurance (Grant et al., 2011). Trans people report low rates of cancer screening, particularly for gendered cancers. For example, it has been reported that trans status is associated with a 37% reduction in odds of cervical cancer screening (Peitzmeier et al., 2014), and significantly less likelihood to engage in screening for prostate cancer (odds ratio 1.07) (Ma et al., 2021) and breast cancer (odds ratio 0.47) (Oladeru et al., 2022), in comparison to cisgender people. This is primarily due to previous negative experiences during screening and reluctance to attend gendered health services (Connolly et al., 2020; Lombardo et al., 2022). Trans people may be excluded from screening reminders or invitations to screen for relevant gendered cancers because of the ways their gender is listed in medical records (Connolly et al., 2020). This is compounded by a pervasive silence and invisibility of trans people in cancer awareness campaigns (Braun et al., 2017; Deebel et al., 2017). These delays and avoidance of care may lead to cancers being undetected or diagnosed at a later stage. In combination with behavioral risk factors and medical comorbidities (Boehmer et al., 2020; Grant et al., 2011), disruptions to care may contribute to poorer outcomes amongst trans people with cancer.

Recent qualitative studies have focused on the psychosocial aspects of cancer survivorship in relation to embodiment and gender affirmation. Identification as trans (binary or non-binary), and the process of gender affirmation, has been described as a process of “becoming,” “home-coming,” and “closure” (Prosser, 1998, p. 90) wherein gender dysphoria is replaced by gender euphoria (Benestad, 2010). Gender dysphoria is defined as a complex and nuanced experience of suffering in response to internal and external stimuli (such as being misgendered) which produce a disconnection between one’s sense of self and the external perception of the self by others (Austin et al., 2022). Gender euphoria involves positive feelings in response to the affirmation of one’s body or gender identity, including comfort, confidence, certainty, satisfaction, and joy (Austin et al., 2022). International studies report that between 42 and 61% of trans individuals use hormonal interventions to affirm their gender, a more common practice for trans women than trans men or non-binary people (Callander et al., 2019; Grant et al., 2011; Scheim & Bauer, 2015). For instance, 20% of trans women in a national US survey had undergone gender affirmation surgery (Grant et al., 2011). Medical transitioning through hormones or surgery has been described as a “restorative” practice (Prosser, 1998: 83), restoring the reality of a person’s somatic experience of gender (Aizura, 2006). This is generally reflected in positive mental health, including reductions in depression, anxiety, and suicidal ideation among trans people (Glynn et al., 2016; Hughto et al., 2020), with gender affirmation often facilitating connection and validation from others (Erich et al., 2008; Peters, 2018).

Embodied changes that occur as a result of medical interventions to affirm gender, such as growth of facial hair or chest surgery, have meaning at a material and discursive level (Ussher et al., 2022b), with the social interpretation of embodied changes central to intrapsychic experience (Dozier, 2005). There is some evidence that cancer treatment may heighten gender dysphoria and disrupt gender affirmation processes (Squires et al., 2022; Taylor & Bryson, 2016). Cancer of the uterus is constructed through gendered discourses such as a “woman’s cancer,” which may be discordant with trans men and non-binary embodiment and identity for those presumed female at birth, resulting in distress (Taylor & Bryson, 2016). Experiences of gender dysphoria for trans men have also been reported about cervical cancer screening, with the need “to focus on an essentially female part” during cervical screening described as “incredibly upsetting” (Johnson et al., 2016, p. 460). Equally, mastectomy following breast cancer may be perceived to “erase” the experienced femininity of trans women and non-binary people presumed male at birth (Taylor & Bryson, 2016). However, removing ovaries, uterus, or breasts can be gender-affirming for trans men (Bilash & Walker, 2022) or non-binary people presumed female at birth (Bilash & Walker, 2022; Taylor & Bryson, 2016). Several studies have indicated that trans and cis lesbian, bisexual or queer (LBQ) patients have begun to embrace the choice to forego breast reconstruction after mastectomy (Brown & McElroy, 2018; Rubin & Tanenbaum, 2011; Wandrey et al., 2016). A bilateral mastectomy can facilitate an alignment between gender identity and embodiment for trans men and non-binary people presumed female at birth and be medically similar to gender-affirming “top surgery” (Taylor & Bryson, 2016). As a genderqueer participant from Brown and McElroy’s (2018) study explained after making this treatment choice, “I feel like I now have a body that fits me” (p. 411). Equally, a genderqueer participant described their loss of ovaries following cancer as a “godsend” (Alpert et al., 2021, p. 2554).

Interactions with oncology healthcare practitioners can influence how trans people navigate the impact of cancer on embodiment and identity (Alpert et al., 2021; Kerr et al., 2021; Squires et al., 2022). There is evidence that trans men, non-binary people presumed female at birth and LBQ cis women face considerable pressure to undergo breast reconstruction after mastectomy, from both survivor organizations and within the medical system (Brown & McElroy, 2018; Rubin & Tanenbaum, 2011). Oncology healthcare practitioners have been reported to adopt gender binary and heteronormative assumptions, stigmatizing trans people with cancer (Alpert et al., 2021), or behaving in an overtly hostile and prejudicial manner (Ussher et al., 2022e). Many healthcare professionals (HCPs) receive no training on the care of lesbian, gay, bisexual, transgender, queer or intersex (LGBTQI) patients, with specific self-deficits in an objective and self-perceived knowledge of the cancer-related needs of trans patients reported by HCPs (Banerjee et al., 2018; Schabath et al., 2019; Ussher et al., 2022d). Lack of coordination between gender affirming care and cancer care has been reported by trans cancer patients, with implications for cancer survivorship experiences (Alpert et al., 2021; Squires et al., 2022). Urinary incontinence following gender-affirming surgery involving vaginoplasty can be exacerbated by cancer treatment and may also impact bowel continence (Jiang et al., 2019). Dilation of the neo vaginal canal to prevent stenosis may also be impacted by vaginal dryness following cancer surgery (Ingham et al., 2018). Some cancers are treated using hormone therapies, while others are hormone-sensitive, in both cases potentially interacting with hormones used as part of gender affirmation. Cancer treatment decisions may thus have significant and enduring impacts on trans cancer survivors’ embodiment and identity (Squires et al., 2022; Taylor & Bryson, 2016) and complicate the ability to have gender affirming surgery following cancer treatment.

To date, trans cancer survivorship research has primarily focused on small samples of adults with breast or gynecological cancer (Bilash & Walker, 2022; Bryson et al., 2018; Squires et al., 2022; Taylor & Bryson, 2016). Recent systematic literature reviews highlight the need to understand the complexity of LGBTQI experience of cancer, including people who are trans, across a range of cancer streams, ages and sexualities (Clarke et al., 2019; Griggs et al., 2017; Pratt-Chapman et al., 2021; Quinn et al., 2015). There is also a need to examine the perspectives and experiences of informal cancer carers who are trans, a group who are often invisible within LGBTQI cancer research and care (Arthur & Kamen, 2018; Kamen et al., 2022). Cis LBQ partner-carers have reported being excluded from consultations with HCPs (Margolies & Scout, 2013), and not offered supportive services typically offered to heterosexual couples (Kamen, 2018), with disclosure of sexuality to HCPs associated with distress (Boehmer et al., 2005). The impact of informal cancer caring on gendered identity and sexual embodiment has been identified in the general cancer population (Gilbert et al., 2010; Ussher et al., 2013). There is no previous research on the impact of informal cancer caring on the embodiment and identity of trans people.

To address these gaps in the research literature, the present paper aims to examine the experiences of embodiment and identity of trans people with cancer and their informal carers, across a range of ages, tumor types and sexualities. This analysis is part of a broader mixed methods study on LGBTQI cancer and complements quantitative analysis, which found significantly higher levels of distress and discrimination in cancer care, and greater impact of cancer on LGBTQI identity and gender identity, in trans participants compared to cisgender lesbian, gay, bisexual, and queer (LGBQ) participants (Ussher et al., 2022a).

The research questions we address in this paper are: How does cancer and cancer treatment impact the embodiment and identity of trans people with cancer and trans cancer carers? How do interactions with healthcare professionals (HCPs) influence the negotiation of trans embodiment and identity in the context of cancer?

## Method

### Study Design and Theoretical Framework

This study was part of the broader mixed methods Out with Cancer project (Power et al., 2022; Ussher et al., 2022a, d, e). The project examined LGBTQI experiences of cancer from the perspectives of LGBTQI patients, their caregivers, and healthcare practitioners to inform LGBTQI inclusive cancer care. This paper presents the qualitative analysis of open-ended survey responses, interviews and a photo-elicitation activity related to trans cancer survivors and carer experiences of embodiment and identity. By embodiment, we mean the experience of living in, perceiving, and experiencing the world from the location of our gendered bodies and how our social environments “enter into and become entangled with our bodies” (Tolman et al., 2014, p. 761). By identity, we mean a sense of self as a person, which can include gender identity (e.g., male, female and non-binary) and sexual identity (e.g., gay, lesbian, queer, asexual, pansexual, straight) (Diamond et al., 2011).

The project is informed by an intersectional theoretical framework, which acknowledges that individuals inhabit multiple interconnected social identity categories, such as gender, sexuality, cultural background and age (Crenshaw, 1991). These identity categories are embedded in systems of social stratification, associated with power inequalities (Hawkey & Ussher, 2022; Marecek, 2016), and influence social practices, health and well-being (Hankivsky et al., 2010). Central to intersectionality is the practice of stakeholder involvement, described as integrated knowledge translation (iKT) (Graham et al., 2006), a dynamic, collaborative process between researchers and knowledge users to achieve actionable research outcomes, which guided the study design, data collection, analysis and dissemination.

Following principles of iKT, a steering committee comprising LGBTQI people with cancer, cancer clinicians, and representatives from LGBTQI health and cancer support organizations were actively involved through co-design in all stages of the study. This involved collaborative development of the research proposal and funding application; co-funding of the research between partner organizations and the national government funding body (Australian Research Council); collaboration of the steering committee in recruitment, coding of data, analysis of themes and production of manuscripts; and translation of findings into resources for patients, carers and HCPs. The core research team (JU, RP, KA, JP, AH, SS, CE) conducted the day to day data collection and analysis, meeting with the other chief investigators (CIs) and the steering group face to face or on zoom on a three monthly basis on average. The steering group were also in contact with the core research team to provide additional advice and support when it was needed, such as recruiting hard to reach groups, developing coding frames, or when commenting on multiple drafts of analysis plans and papers. Expectations of the core group of researchers, broader group of CIs and steering group were discussed at the beginning of the project, including demands on time and involvement in dissemination of findings. Any disagreement between the members of the group were dealt with through dialogue and discussion until consensus was reached. Discussion between the researchers and the steering group facilitated reflexivity (Berger, 2015), a critical evaluation of how our positions as LGBTQI people, clinicians, researchers, and/or cancer survivors influenced the research process and outcome. Most of the core research team, several of the CIs, partner organization members and all end users with lived experience of cancer were LGBTQI. The remaining team members were cisgender heterosexual clinicians, researchers, or organizational partner members with experience in oncology or embodiment theory.

### Participants and Procedure

Participants were eligible for this study if they: (a) had been diagnosed with cancer, had undergone a medical intervention related to cancer risk or had cared for someone with cancer; (b) they or the person they cared for identified as LGBTQI; and (c) were at least 15 years old. Participants were invited to opt into the study through cancer and LGBTQI community organizations, including the study partner organizations, social media (Facebook, Twitter, Instagram), cancer research databases, cancer support groups and LGBTQI community events. Snowball sampling was also used, asking participants to pass the study information to someone they knew who fitted the study criteria. The study was open internationally, although recruitment focused on Australia and other English-speaking countries such as the USA, UK, New Zealand, and Canada. Data were collected between September 2019 and September 2021.

In this paper, we draw on the accounts of 86 participants, including trans cancer survivors (*n* = 63) and trans carers (*n* = 23), who represented a broad range of cancer types and age groups (mean age 47 for cancer survivors, 42 for carers, range 17–72 years) (see Tables 1 and 2). The majority (*n* = 53) self-identified as non-binary, with 18 identifying as trans women, and 10 as trans men. The majority (89.2%) were Caucasian, identifying as lesbian, gay or homosexual (26.7%), bisexual (23.3%) or queer (30.2%), with a minority identifying as heterosexual (9.3%). The majority lived in Australia (64.0%) or the USA (15.1%), in an urban (52.9%), regional (36.5%) or rural (10.6%) location. Seventeen (33%) of the trans participants reported an intersex variation (14 cancer survivors, 3 carers), most of whom had experienced medical intervention to prevent cancer.

**Table 1:** Demographics of trans cancer survivors and carers

Demographic characteristic	Cancer survivors (*n* = 63)	Carers (*n* = 23)
	*M (SD),* range	*M (SD),* range
*Age at time of study* (years)	42.7 (15.3), 17–72	42.5 (16.3), 17–70
	*n* (%)	*n* (%)
*Country*		
Australia	36 (57.1%)	19 (82.6%)
USA	13 (20.6%)	0
UK	6 (9.5%)	2 (8.7%)
New Zealand	3 (4.8%)	1 (4.3%)
Other^a^	5 (7.9%)	1 (4.3%)
*Location*		
Urban	30 (48.4%)	15 (65.2%)
Regional	26 (41.9%)	5 (21.7%)
Rural or remote	6 (9.7%)	3 (13.0%)
*Race/ethnicity*		
Caucasian	54 (90.0%)	20 (87.0%)
Mixed background	5 (8.3%)	2 (8.7%)
Other/unclear background^b^	1 (1.7%)	1 (4.3%)
*Gender*		
Non-binary (assumed female at birth)	26 (41.2%)	15 (65.2%)
Non-binary (assumed male at birth)	10 (15.9%)	0
Trans female	13 (20.6%)	5 (21.7%)
Trans male	8 (12.7%)	2 (8.7%)
Different identity^c^	6 (9.5%)	1 (4.3%)
*Sexuality*		
Lesbian, gay or homosexual	15 (23.8%)	8 (34.8%)
Bisexual or pansexual	17 (27.0%)	3 (13.0%)
Queer	16 (25.4%)	10 (43.5%)
Straight or heterosexual	6 (9.5%)	2 (8.7%)
Different or multiple identities^d^	9 (14.3%)	0
*Intersex variation*		
Yes	14 (22.2%)	3 (13.0%)
No	41 (65.1%)	20 (87.0%)
Prefer not to answer	8 (12.7%)	0
*Relationship status*		
Not in a relationship	17 (36.2%)	5 (27.8%)
Casually dating	4 (8.5%)	2 (11.1%)
Relationship with one other person	21 (44.7%)	10 (55.6%)
Multiple relationships	5 (10.6%)	1 (5.6%)
*Education*		
Less than secondary	2 (3.2%)	2 (8.7%)
Secondary	5 (7.9%)	3 (13.0%)
Some post-secondary	9 (14.3%)	2 (8.7%)
Post-secondary	47 (74.6%)	16 (69.6%)

^a^Other countries: For cancer survivors, Austria, Canada, Denmark, Russian Federation, Serbia (*n* = 1 each). For Carers, Germany (*n* = 1)

^b^Other backgrounds: For cancer survivors, Latinx (*n* = 1). For carers, Jewish (*n* = 1)

^c^Other gender identities: For cancer survivors, not clearly specified (*n* = 3); non-binary (no sex specified), trans (not further specified), questioning (*n* = 1 each). For carers, non-binary (no sex specified) (*n* = 1)

^d^Other sexualities: For cancer survivors, transgender (*n* = 3), asexual/demisexual (*n* = 2), lesbian and queer, not sure, prefer not to say (*n* = 1 each)

**Table 2:** Cancer characteristics of trans cancer survivors and carers

Cancer characteristic	Cancer survivors (*n* = 63)	Carers (*n* = 23)
	*M (SD),* range	*M (SD),* range
*Age at diagnosis* (years)	37.0 (15.4), 8–69	33.9 (15.7), 9–64
	*n* (%)	*n* (%)
*Cancer diagnosis (first)*		
Brain	3 (4.8%)	2 (8.7%)
Breast	7 (11.1%)	8 (34.8%)
Colorectal	1 (1.6%)	4 (17.4%)
Head/neck	2 (3.2%)	3 (13.0%)
Leukemia	3 (4.8%)	1 (4.3%)
Prostate	5 (7.9%)	0
Skin	6 (9.5%)	0
Uterine	4 (6.3%)	0
Other^a^	11 (23.9%)	4 (17.4%)
Not sure, unknown or multiple	4 (6.3%)	1 (4.3%)
*Cancer stage*		
Localzed	30 (65.2%)	10 (43.5%)
Regional	9 (19.6%)	5 (21.7%)
Distant/metastatic	2 (4.3%)	5 (21.7%)
N/A	1 (2.2%)	0
Not sure or unclear	4 (8.7%)	3 (13.0%)
*Subsequent cancers*		
Recurrence	12 (26.1%)	5 (21.7%)
New primary cancer	4 (8.7%)	3 (13.0%)
*Treatment status*		
No treatment yet	4 (8.7%)	2 (8.7%)
On active curative treatment	4 (8.7%)	3 (13.0%)
On maintenance treatment	11 (23.9%)	2 (8.7%)
In remission/completed treatment	25 (54.3%)	7 (30.4%)
Receiving palliative care (no further active treatment)	0	1 (4.3%)
Deceased	–	8 (34.8%)
Not sure, unclear or multiple	2 (4.3%)	0
*Carer’s relationship to cancer survivor*		
Partner		10 (43.5%)
Friend		4 (17.4%)
Child		3 (13.0%)
Parent		2 (8.7%)
Sibling		2 (8.7%)
Other family		2 (8.7%)

^a^Other diagnoses: For cancer survivors, bladder, ovarian, sarcoma, testicular (*n* = 2 each); anal, kidney, lymphoma, pancreatic, thyroid (*n* = 1 each). For carers, bladder, cervical, lung, lymphoma (*n* = 1 each)

Participants took part in a three-stage study. All participants completed an online survey. A subset of survey participants, 15 survivors and 7 carers, completed a 60-min interview to investigate their experiences in greater depth. Six survivors and 2 carers completed an additional photo-elicitation activity. Sample size for interviewees was determined by principles of information power (Malterud et al., 2016), no new material appearing in three successive interviews.

### Materials

#### Survey

The survey comprised a series of closed and open-ended measures. Full details of the survey are described in detail elsewhere (Ussher et al., 2022a). This paper focuses on responses to open-ended questions on embodiment, identity, and interactions with healthcare professionals associated with embodiment and identity. Following closed ended measures about the impact of cancer on trans identity (Has cancer impacted on your feelings about your gender identity (e.g., as a man, woman, transgender, non-binary or gender fluid person? Since your cancer experience have you had concerns about changes to your body?) and interactions with healthcare practitioners (If any of your cancer healthcare professionals are not aware you are transgender, non-binary, or gender fluid, why is this?), participants were asked “Is there anything you would like to tell us about this issue?”

#### Semi-Structured Interview

Semi-structured one-to-one interviews, using a conversational style, were undertaken to explore subjective experiences in-depth. Audio-recorded interviews were conducted over the telephone or using video-conferencing software. Participants were asked about their experiences of cancer, including interactions with HCPs, decision-making pertaining to disclosure of their LGBTQI status and the consequences of this for their cancer care; the impact of cancer on their lives, including on their identities and relationships; and experiences of finding support networks and information as an LGBTQI cancer patient.

#### Photo-Elicitation Activity

Interview participants were invited to engage in a photo-elicitation activity. Photo-elicitation is a method in which photographs are used as a stimulus to elicit rich accounts of experience in subsequent interviews (Frith & Harcourt, 2007). Photo-elicitation is a variation of photovoice methods (Wang & Burris, 1997), involving participants taking photographs that reflect surroundings and life experience, facilitating communication with policy makers and other stakeholders (Valera et al., 2009). Situated within a feminist action-research model, photovoice methods facilitate involvement and empowerment of research participants and have been described as an innovative way of working with marginalized people, including LGBTQI communities (Ussher et al., 2022b, c). Following Frith and Harcourt (2007), we used the photographs as a reference point in one-to-one conversations, allowing questions to be asked about participants understanding of the image, the context of the image, and how they make sense of the image in the present moment. Interviews undertaken in conjunction with photographs have been found to yield richer information than that generated by verbal interviews (Samuels, 2012).

Participants were invited to submit three to five photographs that represented their experiences with cancer or cancer care, which were then discussed in a second interview. Written and visual instructions were provided to participants to aid in the photovoice process. Participants used their devices (smartphones, digital cameras) to take photographs and electronically submit them to the research team. All participants provided informed consent for their photographs to be used in analysis and publications, including personally identifying photographs.

### Data Analysis

Reflexive thematic analysis was used to analyze the open-ended survey, interview and photovoice data, as an appropriate method to capture richness across multiple data types (Braun & Clarke, 2019). The interpretation of the photographs by participants was thematically analyzed alongside the verbal accounts. All interviews were electronically transcribed verbatim, and integrity checked for any errors by listening to the audio against the transcript. Through a collaborative process with stakeholder committee members, a subset of interviews was independently read and re-read to identify first-order codes within the patient and carer data sets that represented commonality across accounts, such as “embodied change,” “identity,” “interactions with HCPs,” “difficulties in communication.” Each team member brought suggestions of the first order codes to the meeting and the final coding frames were devised through a process of consensus. This included codes such as “impact on gender affirmation,” “culturally safe care, services and support,” and “disclosure of identity.” Open-ended survey and interview data were coded by two members of the core research team using NVivo. Consistency in coding across codes and coders was checked by the senior member of the team. Coded data were read through and summarized in a tabular format to facilitate identification of commonalities in the data. The codes were then re-organized and grouped into themes focused on experiences of embodiment and identity for trans people and trans carers. Themes were refined through discussion, reorganized and, when consensus was reached, final themes developed. Throughout, the analysis was informed by an intersectional theoretical framework. This acknowledges the interaction and mutually constitutive nature of gender, sexual identity, age and other categories of difference in individual lives and social practices, and the association of these arrangements with health and well-being (Hankivsky et al., 2009). Members of our stakeholder advisory group were involved in developing codes and themes and read and provided comments on the interpretation and reporting of the data. The analysis was revised to incorporate feedback on language and interpretation.

**Fig. 1:**
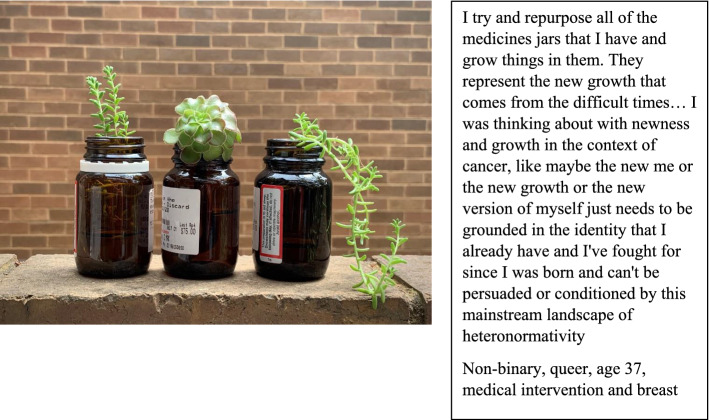
New growth from difficult times

**Fig. 2:**
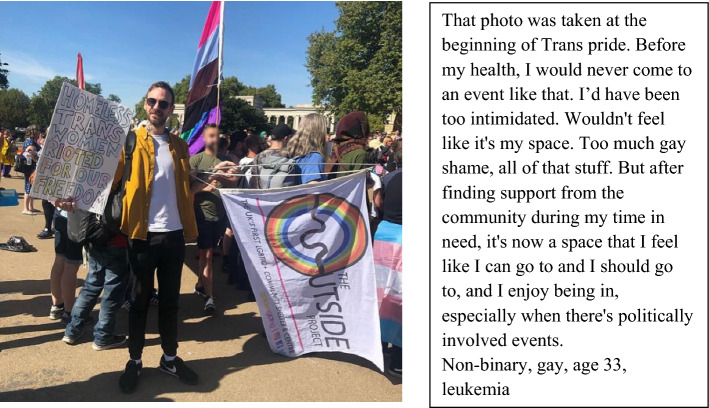
Trans pride

**Fig. 3:**
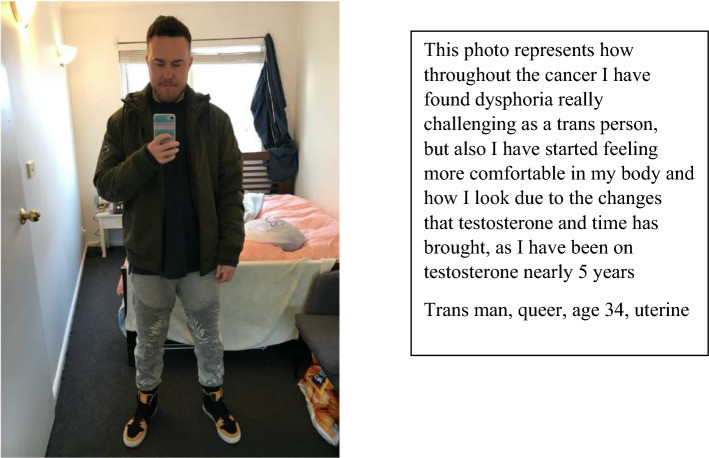
Body dysphoria

**Fig. 4:**
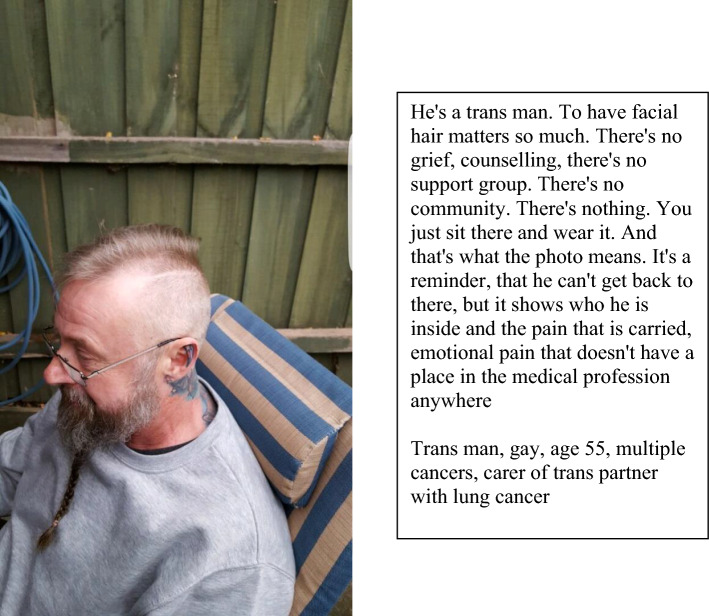
Emotional pain that doesn’t have a place

**Fig. 5:**
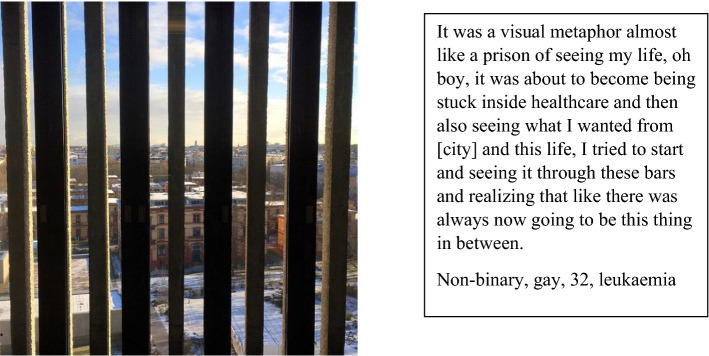
Being stuck inside health care

In the presentation of results, we use the term trans to refer to the participants as a group, reflecting advice from our stakeholder group. When quoting participants, we use their nominated gender descriptor and provide demographic details of age, sexuality and cancer type for longer quotes where participants provided this information. We present the photographs to illustrate the themes within the data based on the accounts given by the participants. A summary accompanies photo-elicitation images (Figs. 1, 2, 3, 4, and 5) in the participant's words.

## Results

### Summary of Themes

We identified three themes: (1) Cancer Enhances Trans embodiment: subthemes—Gender Euphoria Following Cancer Treatment; Cancer Accelerates Decisions about Gender Affirmation; (2) Cancer Erases or Inhibits Gender Affirmation: subthemes—Cancer Disrupts Trans Embodiment; Cancer Caring Interrupts Gender Affirmation; (3) Trans Embodiment Invisible or Pathologized in Cancer Care.

### Cancer Enhances Trans Embodiment

#### “More Connected with my Body”: Gender Euphoria Following Cancer Treatment

For several participants, cancer treatment was “a gift” that served to create a feeling of “relief” or “wholeness,” increasing gender euphoria. The notion of “new growth” and feeling “grounded in identity” after cancer for a non-binary participant is illustrated in Fig. 1.

For many participants, gender euphoria resulted from the embodied “side effects” of cancer treatment, resulting in greater alignment of body and identity. For example, hormonal cancer treatment and chemotherapy that resulted in the cessation of menstruation and changes in body hair had a positive defeminizing effect for several participants. This is exemplified in the account of a 52-year-old trans man with thyroid cancer who received information from HCPs conveying, "that ‘we are sorry to say that one possible unpleasant side effect of chemo is that you may....’was very confusing, as all the ‘negative’ de-feminising side effects caused internal gender euphoria.” Removal of the uterus and ovaries following diagnosis of ovarian cancer can lead to feelings of “relief” and a sense of wholeness: “It was like a million bricks flying off my shoulders when I woke up from surgery. And I didn't have these parts in me anymore. I felt more whole than I had in my entire life” (trans man, straight, age 47, ovarian). He went on to say, “being diagnosed with cancer was actually a gift to me. The surgery alleviated me of some of the parts that were at the core of my gender dysphoria symptoms at the time.” Reductions in erectile function, penis size or testicle size, often accompanied by feminizing bodily changes, are common consequences of prostate cancer treatment, which can be positive and affirming of identity for trans women. As a trans woman taking hormone therapy for prostate cancer told us: “having a shorter and non-erect penis is a positive for me, as is reduced muscle mass and strength and breast sensitivity—at least now I have breasts that are sensitive” (gay, age 63). Reduction in testicle size is a further consequence of hormonal treatment for prostate cancer, which can have positive consequences for trans women, mirroring the impact of gender affirming hormonal therapy (GAHT).

Hormone blockers mean that my testicles, well they're very small now, and I don't care if they stay there because I can wear tighter panties and hide everything, Nothing shows, really….It is good the HRT for being transgender and the treatment for cancer is one and the same, which makes me very happy.

Trans woman, age 63, prostate.

A bilateral mastectomy following breast cancer has similarities to gender-affirming “top surgery”, described as “living flat” or “flattopping”, and can facilitate an alignment between a male or non-binary gender identity and embodiment. Several trans participants described euphoria at the loss of breasts that they no longer had to hide: “I'm more connected with my gender non-conforming identity. Breasts caused me some dysphoria…clothes fit better now I don't have to hide breasts in them somehow” (non-binary, lesbian, age, 52, breast); “I knew I wanted to live flat, so in an 'odd' way, cancer allowed me to achieve the flat chest and breastless body I wanted. I'm probably more connected with my body” (non-binary, queer, age, 37, multiple cancers). However, the aesthetics, mobility and recovery resulting from mastectomy without reconstruction is often very different from top surgery. As the non-binary participant who felt more “connected” with their body explained:

[double mastectomy] itself is a very different procedure. They remove every bit of breast tissue...You don't get pecs, you don't get nipples—I don't have nipples. You don’t get to have a body that resembles any mainstream body. You just have to take whatever comes out at the other end of that surgery, and for some people, that means they have lumpy bits here and there or dog ears or concaved chests, and for other people, that means that they get really flat smooth chests; everyone has a different physical response to mastectomy surgery. And for me it’s been nothing like top surgery...I still can't reach above my shoulders; I can't reach up high or reach the middle of a table or pick up something from the ground easily...[top surgery] is far more recoverable.

Non-binary, queer, age 37, multiple cancers

The outcome of mastectomy without reconstruction for this participant lacks elements of “mainstream” masculinity, suggesting some ambivalence about their embodied change after cancer. For other participants, ambivalence was reflected in relief at the absence of menstruation being tempered by being diagnosed with a “woman’s cancer.” As a trans man told us, “the symptoms both brought me relief (lack of periods) and torment (years of being misdiagnosed, having constant focus on my ‘womanhood’, which had a huge impact on me as a transgender man” (straight, age 38, ovarian). The removal of ovaries was associated with “some grief about the loss of those parts (that) were part of me” for a trans man with ovarian cancer. He went on to say, this “doesn’t affect my masculinity” because “there’s lots of nuances to being transgender” (straight, 47). These accounts demonstrate the complex meanings attached to embodied change after cancer, even when such changes are welcomed.

Gender affirmation facilitated diagnosis of cancer for some participants: “being transgender has saved my life with the cancer because when I had a blood test to go start going on HRT, the PSA was up to seven. And they decided that I needed to be investigated” (trans woman, age 63, prostate).

One of my skirts had actually broken the skin on a mole on my knee. If I hadn't been squeezing myself into tight skirts and starting HRT and putting on weight, I might never have found it because I probably wouldn't have ever gone to get a skin check

Trans women, bisexual, age 30, melanoma

These experiences reinforced a positive association between gender affirmation and cancer, which was able to be successfully treated due to early intervention.

#### “It Has Made Me Want to be More Open”: Cancer Accelerates about Gender Affirmation

For some, a cancer diagnosis precipitated or accelerated the decision to engage in gender affirmation, leading to self-identification and coming out to others as trans. For example, for one participant, being told that they needed a mastectomy “was the moment that the genie came out of the bottle,” allowing them to acknowledge their true identity as a trans man because of the “utter relief and awareness they [breasts] so long ago should have been off” (trans man, gay, age 55, multiple cancers). Other participants said, “I feel more non-binary” after cancer (non-binary, queer, age 58, breast); and “My witnessing all the intervention possibilities has given me courage to embrace gender-affirming surgery and hormones” (trans man, queer, age 51, thyroid). Cancer can also result in reflection on what is important in life and allow individuals to “come to terms with” their trans identity and be “vocal about it.”

But then, when I got diagnosed with cancer, it made me…focus on that a lot more, about…actually, who am I, and how do I want to continue, and… at what point am I going to ignore these parts of myself. So, coming to terms with it's been a recent thing anyway. Or- not coming to terms with it but being vocal about it.

Non-binary, gay, age 32, leukaemia.

Support from the trans community during cancer could facilitate gender affirmation and greater openness about trans identity in the context of cancer, as illustrated in Fig. 2.

Some participants expressed regrets at not affirming their gender prior to cancer. For example, a 40-year-old non-binary bisexual participant told us that cancer “added to my resolve to be a bit more visible, a bit more open… and I look back and I think I probably could have done a lot more had I been more visible and open for longer.” Another participant said:

I've had strong feelings of regret that I didn't live as myself sooner and medically transition sooner. In particular, the transition-related surgery I knew I wanted would have prevented my cancer entirely. Cancer has made me want to be more open about being a nonbinary trans person

Non-binary, queer, pansexual, age 32, testicular.

Rejecting gender expectations of others could be part of this process, with cancer serving to liberate anger: “It (cancer) made me angrier about people assuming that I fit a female template and can live that way. I think the anger was ultimately good for me” (Non-binary, queer, intersex, age 48, medical intervention).

Informal cancer carers who are trans can also experience a realization of their trans identity or have the confidence and impetus to engage in gender affirmation, because of their cancer caring experience. A trans woman told us that her “transition [occurred] through that period” of caring for her partner with breast cancer, following a discussion of “how we would spend our last day. And that led to me confessing to her that I'd rather spend the last day of my life authentically” (trans woman, bisexual, intersex, age 59, carer of partner with breast cancer). A non-binary carer told us that they realized the “disconnect” with their body and their “suppressed” feelings about gender affirmation when they witnessed their partner’s distress at the loss of her breasts:

Witnessing her going through that kind of made me realize my disconnect with my own body.…I guess it just kind of like triggered me to actually connect just those feelings I’ve had for a really long time. You do what you can to suppress anything that's too difficult to talk about. And it just kind of triggered a journey for me to start processing some stuff which I never processed before….I think in new situations I feel like I can like present who I am now.

Non-binary, queer, age 32, carer of partner with breast cancer

Realizing what is important in life, and becoming immune to the reactions of others, can be part of this cancer precipitated gender affirmation process, as a participant whose partner died from breast cancer told us: “I feel more happy and confident to be myself after experiencing caring for my partner and her loss. I sweat the small stuff less and really am not bothered about what people think. It’s not their business” (non-binary, lesbian, age 49, carer of partner with breast cancer).

### Cancer Erases or Inhibits Gender Affirmation

#### “Terrible Gender Dysphoria”: Cancer Disrupts Trans Embodiment

For other trans participants, diagnosis and treatment for cancer had a detrimental effect on embodiment and identity, disrupting the euphoria that they had experienced during gender affirmation. For example, a trans woman with melanoma (lesbian, age 40), told us “it (diagnosis) came so soon after I began my transition and I was happy for the first time in my life and then in the blink of an eye, that brief feeling of elation was cut short. That sucked.” Several participants reported that cancer diagnosis heightened gender dysphoria, due to the cancer being related to “sex assigned at birth” and to organs that felt alien or were not wanted. As a trans man diagnosed with uterine cancer told us:

The type of cancer I have had, which relates to my sex assigned at birth, can be distressing and is hard to cope with and brings about a lot of dysphoria. It’s more having a stupid cancer associated with my organs I don't want and were taken out but still I have to deal with when I just want to forget they even existed and don't want to think about that stuff ever

Trans man, queer, age 34, uterine.

These feelings of dysphoria were somewhat ameliorated by the impact of GAHT on the participant’s body and sense of self, illustrated in Fig. 3.

Body changes resulting from cancer treatment may also impact upon embodiment and identity and create dysphoria. For example, a trans man told us of the trauma of losing his facial hair during chemotherapy, which resulted in him being “misgendered as a woman,” contrasting the support offered to cisgender women with lack of recognition of his loss:

There was no support, recognition or understanding of the impact of losing the beard hair being a trans man. I had to sit across from women getting fussed over and getting head cool pack things to try to stop their hair loss. There was no recognition or help for my loss, so linked to my identity

Trans man, gay, age 55, multiple cancers.

This participant also cared for his trans male partner who had lung cancer. He depicted his partner’s grief following the loss of facial hair, and lack of recognition within the health system of this grief, through a photograph taken before chemotherapy (Fig. 4).

Weight changes can also lead to misgendering and dysphoria. A trans man with ovarian cancer explained how his masculinity was challenged by treatment-related weight gain: “the lymphedema causes me gender dysphoria because it feminizes my abdomen/hips” (age 47, heterosexual, ovarian). He also told us that “the compression garments push down my packer to the point it causes me gender dysphoria.” Another participant was distressed about the “ultra-femme pink” color of compression garments, contrasting the feminizing impact of lymphedema with masculinizing “top surgery.”

If top surgery is more like masc-presenting, with lymphedema is like ultra-femme because you get swollen and you get curvy and you get lumps in different areas. And all of the lymphedema stuff is like ultra-femme, it's like pink or tan

Non-binary, queer, 37, multiple cancers.

Several trans women reported distress associated with mastectomy. For example, a trans woman who had had a bilateral mastectomy due to family risk of cancer felt regret at the loss of her “natural breasts.”

As a trans woman, I was very pleased that my body developed breasts naturally at around age 45. I was not taking hormones. I wanted an orchidectomy, but when I contacted specialists, I was advised that due to very high cancer risk due to family history, that I had to have a bilateral mastectomy. In hindsight, I wish that I hadn't had the mastectomy. Having natural breasts was a big part of my female identity.

Trans woman, unsure sexuality, age 56, intervention to reduce cancer risk

A non-binary participant who had a mastectomy to prevent cancer described “the emotional toll of having a completely different body to have body dysmorphia about” following “double mastectomy and reconstruction.” They described “being put in lots of spaces for women” which made them “feel like a fraud” (Non-binary, queer, age 38, medical intervention to reduce cancer risk).

Hormones used during fertility preservation following cancer diagnosis can have a material and psychological impact on experiences of gendered embodiment. A trans carer reported experiencing “catastrophic depression” and “terrible gender dysphoria” because of his engagement in fertility preservation treatment some years ago to preserve an embryo. He told us, “when I was given steroids to provoke ovulation, I attempted suicide because of how fucked up my trans brain responded to even more oestrogen” (trans man, queer, age 53, carer of partner with thyroid cancer). The impact of hormonal treatments on sexual embodiment could also detrimentally affect sense of self. For example, a non-binary queer breast cancer survivor, age 49 said that “tamoxifen drew me into menopause and I wasn’t feeling very sexual… I mourn that part… it wasn't for me about how I looked it was just about how I feel internally.”

#### “I Put It on Hold”: Cancer Caring Interrupts Gender Affirmation

Caring for a partner or family member who has cancer can interrupt the process of gender affirmation for trans people. This demonstrates that the impact of cancer on gendered embodiment goes beyond the material changes resulting from cancer treatment. Caring took up all of their emotional energy, and “dealing with” gender affirmation was “put on hold” or “put to the side,” because “it just wasn't something that either of us were able to even consider looking at and talking about… it was very much a focus on you just had to survive” (non-binary, queer, age 38, caring for partner with breast cancer). This participant also reported guilt about their desire to engage in medical gender affirmation which mirrored the cancer treatment their partner had undergone:

Even contemplating top surgery for me was something that I still feel immense guilt over. My partner has had a mastectomy to save her life due to the cancer. And that was something out of her control. So then, in my head it's like, well, I'm opting to have the same part of my body removed. But there's nothing wrong with my body to have it removed.

Gender affirmation and coming out in relation to sexuality were often interconnected and disrupted by cancer caring. A 20-year-old bisexual non-binary person caring for their father with brain cancer told us: “I was still discovering my sexuality when I was a carer and felt like I didn't have the emotional energy to deal with it so tried to think about it as little as possible.” As a result, they said they were “not out when I was caring.” A 38-year-old queer non-binary person caring for their grandmother with bowel cancer felt “embarrassed” and “ashamed” in hospital settings because of looking “unusual” due to “being shaved up, having grown facial hair.” They dealt with these feelings by stopping the GAHT which they had been taking for four years:

While I was a carer that role came before my own needs as non-binary; I stopped taking hormones and presented as more feminine. As a carer I was less open about being non-binary as I did not want it to affect me or my grandmother's needs.

This had negative intrapsychic consequences, as having “to feel that I had to pretend to be someone else was upsetting and stressful,” and the abrupt cessation of testosterone meant they were “more tearful, anxious and depressed.” After the caring role had ended, they said, “I didn't actually know how to find myself again.” Disrupted gender affirmation also had consequences in terms of isolation and support. They continued: “I felt more isolated from the LGBTIQ + community while I was a carer. I did not feel supported and also stopped interacting with this part of the community.”

### “A Complete Lack of Understanding”: Trans Embodiment Invisible or Pathologized in Cancer Care

Interactions with HCPs were key to negotiating embodiment in the context of cancer for trans patients and carers and could exacerbate distress and dysphoria. Invisibility within a cisgenderist health system, due to information on sexuality, gender and intersex status not being collected, was commonly reported: “The lack of accurate gender questions (current gender AND sex assigned at birth) and questions about intersex status has a direct impact on our safety and quality of care” (non-binary, queer, age 38, cervical); “It matters that cis-heterosexuality remains the norm and the default” (Non-binary, queer, age 33, testicular). This lack of acknowledgement could result in embodied dysphoria not being “seen” by HCPs.

All of the trans dysphoria was in play but un-named for the cancer treatment. We (my husband and I) would have had a much less traumatic time if my psychological distress had been "seen" or diagnosed as body and gender dysphoria

Trans man, queer, age 52, thyroid

Being treated in a gendered hospital system which is discordant with a person’s trans identity can be “very upsetting,” as a trans man with uterine cancer treated in a women’s hospital told us: “Sometimes when I am contacting the hospital and speak with staff on the phone they dismiss me or tell me I have the wrong hospital” (queer, age 34). Experiences of having little agency and feeling “stuck inside health care” as a trans person are illustrated in Fig. 5.

A lack of understanding from HCPs of the meaning and impact of cancer treatment about trans embodiment was common. For example, a non-binary prostate cancer survivor told us there was “no alternative offered to radical prostatectomy, no consideration of my transgender status”. This had implications for their ability to medically affirm their gender, which left them feeling “cheated.”

To be honest, if they had been more understanding and listened to me, I could have had a bottom transition operation. Rather than being left without any ability [for medical gender affirmation] and no chance of transition due to the extensive damage done.

Non-binary, age 68, prostate and pancreatic.

A breast cancer survivor who told “a team of female doctors” that they would “rather have [my breasts] removed, they really aren’t important to me” said they were met with the HCP response, “why would a female not want to have breasts? Let’s collectively gawk at the patient until they agree to save them” (non-binary, bisexual, age 37, breast). This resulted in the participant feeling “it’s never going to be safe” and “why couldn’t they just listen to me?” Others talked of being pathologized when discussing treatment. For example, the decision of a non-binary participant (queer, age 37, medical intervention and breast) to have a mastectomy without reconstruction was positioned as a “dysphoric response” by their breast cancer surgeon, who initially refused to do the surgery as a result. Conversely, this same participant was referred to alternative surgeons who reportedly wanted a “DSM diagnosis [of gender dysphoria]” before they would agree to operate without reconstruction.

Lack of HCP knowledge about intersex embodiment led to absence of information and unmet needs, as well as inappropriate heteronormative assumptions about sexuality, for a non-binary participant.

It was difficult for me to get appropriate advice relevant to my genital differences. I didn't suffer any further damage as a result, but it added to the stress of the procedure. I also found that advice given assumed I would be heterosexual and having PIV [penis in vagina] sex.

Non-binary, queer, age 48, intersex, medical intervention to prevent cancer

The lack of understanding from HCPs of the interaction between GAHT and cancer treatment was also experienced, which had implications for treatment delivery and efficacy. For example, a trans man told us:

It is written on my file, but staff do not understand what it means. i.e., have I already transitioned or do I want to?… It is a continual issue when blood tests are read and interpreted against male or female ranges. After chemo and steroids, the bone density clinic had trouble working out what a normal reading would be.

Trans man, gay, age 55, multiple cancers and carer for partner with lung cancer

Conversely, several trans cancer survivors talked about everything in their health being attributed to trans embodiment and identity regardless of the reason for their visit to a HCP: “When you say you’re trans, everything is about being trans, it doesn't matter if you sprained your bloody ankle, everything is because you’re trans” (trans man, gay, age 55, multiple cancers); “I’m really tired of everyone assuming things are caused by hormones, and that being the #1 thing everyone jumps to when they think of trans cancer patients” (non-binary, queer, age 26, ovarian). Others experienced unnecessary or invasive questions about trans embodiment or identity from HCPs that were unrelated to the reason for their visit. For example, a trans man frequently reported: “being asked lots of intrusive questions about being trans” (trans man, queer, age 34, uterine). Some participants felt that they were “not "trans" enough” as a non-binary person, “and if I was to share the information, a doctor would not understand if I was not seeking to transition into a woman” (non-binary, gay, age 35, leukaemia); or told us “The medical profession is always looking for a binary. They're always looking for “Are you a trans man, trans woman-?” They don't understand the myriad of spaces in between” (non-binary, gay, age 32, leukaemia). The ubiquitous use of the color pink in breast cancer information can also be alienating for trans people: “Cancer care is intrinsically gendered… the logo of the pink woman is oppressive” (non-binary, lesbian/queer, age 38, multiple cancers). For trans people of color, feelings of exclusion were associated with the intersection of cultural background, gender, and sexuality, as a participant said to us “It's all really white, and white Australian. My partners have not always been white, and they felt actively excluded from all of the materials I brought home for their sexuality, gender and race” (non-binary, lesbian/queer, age 38, multiple cancers).

Lack of understanding on the part of HCPs, combined with past experiences of hostility and prejudice within health care, can lead to non-disclosure of trans identity or modification of appearance as a protective mechanism. As one non-binary queer breast cancer survivor, age 39, told us “I was very scared about my treatment if I told anyone. I already look alternative and even had a normal hair cut when I knew I had surgeries coming up”. Non-disclosure served to both “avoid confrontation” and “leverage the system” for trans participants. It is notable that accounts of non-disclosure to avoid HCP hostility are predominately from non-binary participants, as was the case with non-binary cancer carers. For example, the experience of a non-binary cancer survivor who said “I ride off the privilege of my gender fluidity constantly in order to grin and bear it, and deal with the cis-normativity that it takes to avoid that aspect of discrimination” (queer, age 37, breast) contrasts with that of a trans man with ovarian cancer, who told us “You can't hide behind just not telling them [you’re trans]” (age 48, straight). Fears of confrontation are based in reality, with hostile interactions with HCPs reported by a number of trans participants. For example, participants told us: “while in hospital I heard them talking about me as if I was a freak being described as high risk need for extra care” (non-binary, age 67, pancreatic and prostate).

I had one therapist in the past….that told me I was unhappy because I wasn’t living according to god’s will. It’s why I hid who I am [in cancer care] because I was so worried about my treatment by others (non-binary, queer, age 39, ovarian).

Experiences of HCP discrimination can have a direct impact on trans identity and embodiment:

The discrimination I have experienced from health professionals during my cancer care has reduced my ability to be proud of who I am. This discrimination prevents me from help seeking for my current maintenance care. There is so much transphobic/cisnormative talk between health workers and staff, I overhear it between them and it feels horrible

Non-binary, lesbian/queer, age 38, multiple cancers

In combination, these accounts suggest that HCP's lack of knowledge and experience of trans embodiment and identities, and exclusion of trans people in cancer information, potentially compromises the quality and sensitivity of care received by trans people with cancer, which has implications for their engagement with cancer care and associated psychological well-being.

## Discussion

The findings of this study demonstrate that trans embodiment and identity, as well as the process of gender affirmation, may be disrupted by a cancer diagnosis, cancer treatment or informal cancer caring. This provides insight into the higher level of distress and greater impact of cancer on trans cancer survivors compared to cis LGBQ survivors (Ussher et al., 2022a). Conversely, cancer and cancer treatment can positively impact the embodied identity and lives of some trans people, despite the anxiety and strain of negotiating medical procedures. However, if HCPs operate within a cis-heteronormative framework and do not understand the meaning of embodied change following cancer treatment for trans individuals, these positive benefits may not be realized. Rather than experiencing feelings of gender euphoria, in public institutional spaces like hospitals, trans people will need to keep self-policing or “splitting” their external presentation and internal sense of self in these spaces (Ussher et al., 2022e).

It is widely recognized that benefit finding is a common consequence of cancer diagnosis (Pascoe & Edvardsson, 2013). Our study identifies benefit finding unique to trans people with cancer. Cancer can accelerate the decision to engage in gender affirmation, facilitating engagement and support from trans communities (Power et al., 2022). Feelings of euphoria following the removal of body parts that are incongruent with gender identities, or the impact of hormonal cancer treatment on secondary sex characteristics, echo the findings of previous research (Alpert et al., 2021). Equally, our findings confirm reports that many trans and cis LBQ cancer patients forego breast reconstruction after mastectomy (Brown & McElroy, 2018; Rubin & Tanenbaum, 2011; Wandrey et al., 2016), an experience that is medically similar to gender-affirming “top surgery” (Taylor & Bryson, 2016). However, it is important to recognize, as Foster (2021) argues, “a mastectomy can be a part of top surgery, but not every top surgery is a full mastectomy.” The sculpted body that follows gender-affirming top surgery may look very different from the body that undergoes a double mastectomy to treat cancer, as was evident in participant accounts in the present study. Thus, deciding to “go flat” following a cancer diagnosis may produce some ambivalence about gendered embodiment. Equally, mastectomy following breast cancer may be perceived to “erase” an individual’s experience and expression of gender nonconformity (Taylor & Bryson, 2016), or affect the experienced femininity of trans women and non-binary people presumed male at birth, as reported in the present study. In combination, the intersection of cancer treatment and gender affirmation, at a material, discursive and intrapsychic level, must be considered by HCPs and those providing health information for trans people with cancer.

Trans populations report significantly higher rates of depression, anxiety, and suicidal ideation than cis populations, associated with gender incongruence and minority stress (Clements-Nolle et al., 2006; James et al., 2016). Our findings confirm that cancer treatment can potentially heighten gender dysphoria (Taylor & Bryson, 2016), with distress associated with embodied change following cancer, most notable for those with cancers of highly gendered body parts, such as breasts or reproductive organs. This distress can result from removing body parts congruent with gender or the impact of interventions such as compression garments, which hinder gender expression. This contributes to the higher level of distress and greater impact of cancer on identities reported by trans cancer survivors in comparison to cisgender LGBQ survivors (Ussher et al., 2022a). Our findings of changes to gendered embodiment and identity for trans cancer carers demonstrate that it is not simply the material impact of cancer treatment on the body that impacts trans embodiment (Dozier, 2005). Accounts of gender affirmation being “put aside” as a necessary sacrifice because focusing on the needs of a loved one with cancer reflects the gendered self-sacrifice many informal cancer carers engage in (Ussher & Sandoval, 2008). However, the distress and dissonance associated with postponing or curtailing gender affirmation may compound the many other factors known to be associated with distress for cancer carers (Hagedoorn et al., 2008; Perz et al., 2011), creating greater vulnerability for trans carers. Other carers concealed their trans identity or attempted to pass because of fear of hostility within the health system, as was the case with some cancer survivors, particularly those who were non-binary. These findings confirm previous reports that cis-heteronormative assumptions of HCPs, combined with oncology HCP hostility and discrimination, leads to LGBTQI patient anxiety associated with disclosure of sexual orientation or gender identity (Banerjee et al., 2020; Fish et al., 2019; Lisy et al., 2018; Rose et al., 2017; Ussher et al., 2022e).

Non-disclosure of trans identity is an understandable self-protective mechanism. Trans people who challenge “the normative coercion to perform gender dichotomously” (Peters, 2018) have been described as “border-dwellers” (Pallotta-Chiarolli, 2010), operating in a space in the middle of the gender binary. Non-binary individuals may have a greater ability to be incorrectly perceived as the gender presumed for them at birth than other trans people, who may have no option but to disclose. However, the ability to blend as cis puts non-binary people at greater risk of a diminished sense of self and gender dysphoria (Cole, 2002). It can be associated with a burden of secrecy, feelings of anxiety, invisibility, and frustration through the cancer journey (Lisy et al., 2018). Non-disclosure may thus add to the stress of cancer and result in poor psychological well-being (Durso & Meyer, 2013). At the same time, disclosure of sexual orientation and gender identity (SOGI) status to HCPs has positive benefits, including greater engagement and satisfaction with care, better illness adjustment, and better mental health (Balik et al., 2020; Ruben & Fullerton, 2018).

HCPs' lack of understanding of the meaning of gender affirmation and the interaction of hormonal treatment and cancer treatment has implications for treatment delivery and efficacy (Squires et al., 2022). This lack of awareness of gender affirming care amongst cancer care professionals makes it difficult for trans patients to make informed medical decisions at the intersection of cancer and gender affirming health care, such as negotiating hormone use during treatment (Taylor & Bryson, 2016; Watters et al., 2015), or making agentic decisions about surgery. The pathologization of trans people who embraced mastectomy without reconstruction, as reported in a previous study (Brown & McElroy, 2018), suggests that trans embodiment and identity are being critically evaluated through a psychiatric lens based on cisgenderist assumptions (Winter et al., 2016). Some HCPs also incorrectly attributed everything in the healthcare interaction to the patient being trans, regardless of the reason for their visit to a HCP, a phenomenon previously described as “trans broken arm syndrome” (Knutson et al., 2016). HCPs can also display “inappropriate curiosity,” asking trans patients intrusive personal questions or conducting physical examinations that are unrelated to the presenting condition (Shepherd et al., 2019). These findings suggest that a gender binary is being applied and enforced by some HCPs, even when they may be attempting to be trans inclusive (Winter et al., 2016).

Higher levels of discrimination in cancer care are reported by trans people compared to cis LGBQ people (Ussher et al., 2022a). Cisgenderist healthcare systems, combined with clinicians’ lack of knowledge and experience potentially compromises the quality and sensitivity of care received by trans people with cancer (Puechl et al., 2019); with implications for engagement with cancer care and psychological well-being (Squires et al., 2022; Ussher et al., 2022e). Clinical environments need to be welcoming and inclusive of trans people, through inclusive and affirming online and in-person booking systems, clinic paperwork, treatment or pathology request forms, and inclusive promotional material and website content (TransHub, 2022). There is a need for HCPs to understand trans-affirming practice and be aware of the higher risk of distress for trans cancer survivors and their informal carers, in particular the intersection of cancer with trans identity and embodiment, difficulties in disclosure of identity and the impact of invisibility in health care (Ussher et al., 2022e).

Surveys of cancer health professionals have evidenced self- deficits in an objective and self-perceived knowledge of the cancer-related needs of trans patients (Banerjee et al., 2018; Schabath et al., 2019), with greater gaps in knowledge identified in relation to trans and intersex people, compared to cisgender LGBQ people (Ussher et al., 2022d). This suggests the potential of more unmet needs for those who are both trans and intersex. Only a small minority of oncology HCPs receive formal education/training on the healthcare needs of trans patients, meaning that “brush stroke imagining” was all HCPs have to rely on (Ussher et al., 2022d), even if most agree that such education or training should be mandatory for HCPs (Schabath et al., 2019; Shetty et al., 2016; Sutter et al., 2020). Specific and community-led training in providing trans-affirming cancer care, and the practice of cultural safety, as part of basic communication training and ongoing professional development is essential (Davies et al., 2021). This training should be embedded in medical and allied health degrees, as well as part of ongoing professional development for health professionals (Pratt-Chapman, 2021; Quinn et al., 2020). Such programs can increase HCP confidence, challenge transphobic stereotypes and increase the likelihood of trans patients receiving culturally safe, inclusive and affirming cancer care.

Assumptions about changes in embodiment for people with cancer, including within healthcare settings, are generally underpinned by dominant discourses of binary gender. We need HCPs to be educated about cis-heteronormative assumptions and skilled in trans-affirming practice, with resources and information regarding treatment options specific to trans populations readily available (Sinding et al., 2007). HCPs need to be aware of the meaning of embodied gender markers for trans people, such as facial hair and a flat chest for some trans men (Dozier, 2005), and breasts for some trans women (Ussher et al., 2022c). This will facilitate their understanding of how cancer treatment can threaten or affirm gender affirmation, with implications for the mental health of their trans patients (Hughto et al., 2020). However, meanings attached to embodied change are individual, and many trans people openly seek to challenge the “normative coercion to perform gender dichotomously” (Peters, 2018, p. 11) through identifying as non-binary, as was the case for the majority of participants in the present study. Assumptions cannot be made about the impact of cancer treatment for a generic “LGBTQI” or “trans” population, as is sometimes the case in health information (Pratt-Chapman et al., 2021). Open communication between trans patients and their HCPs, and respect for patient treatment choices, is essential, in order to identify the meaning and impact of embodied changes following cancer and facilitate agency and a positive sense of embodied identity in recovery.

## Data Availability

Data are available from the authors upon reasonable request.
